# Mass mosquito trapping for malaria control in western Kenya: study protocol for a stepped wedge cluster-randomised trial

**DOI:** 10.1186/s13063-016-1469-z

**Published:** 2016-07-26

**Authors:** Alexandra Hiscox, Tobias Homan, Collins K. Mweresa, Nicolas Maire, Aurelio Di Pasquale, Daniel Masiga, Prisca A. Oria, Jane Alaii, Cees Leeuwis, Wolfgang R. Mukabana, Willem Takken, Thomas A. Smith

**Affiliations:** 1 Laboratory of Entomology, Wageningen University Research Centre, Wageningen, The Netherlands; 2 Human Health Division, International Centre of Insect Physiology and Ecology, Nairobi, Kenya; 3 Department of Epidemiology and Public Health, Swiss Tropical and Public Health Institute, Basel, Switzerland; 4 University of Basel, Basel, Switzerland; 5 Knowledge, Technology and Innovation Group, Wageningen University and Research Centre, Wageningen, The Netherlands; 6 Context Factor Solutions, Nairobi, Kenya; 7 School of Biological Sciences, University of Nairobi, Nairobi, Kenya

**Keywords:** Vector control, Mass trapping, Anopheline mosquitoes, Odour-baited trap, Transmission, Clinical malaria, Stepped wedge cluster-randomised trial

## Abstract

**Background:**

Increasing levels of insecticide resistance as well as outdoor, residual transmission of malaria threaten the efficacy of existing vector control tools used against malaria mosquitoes. The development of odour-baited mosquito traps has led to the possibility of controlling malaria through mass trapping of malaria vectors. Through daily removal trapping against a background of continued bed net use it is anticipated that vector populations could be suppressed to a level where continued transmission of malaria will no longer be possible.

**Methods/design:**

A stepped wedge cluster-randomised trial design was used for the implementation of mass mosquito trapping on Rusinga Island, western Kenya (the SolarMal project). Over the course of 2 years (2013–2015) all households on the island were provided with a solar-powered mosquito trapping system. A continuous health and demographic surveillance system combined with parasitological surveys three times a year, successive rounds of mosquito monitoring and regular sociological studies allowed measurement of intervention outcomes before, during and at completion of the rollout of traps. Data collection continued after achieving mass coverage with traps in order to estimate the longer term effectiveness of this novel intervention. Solar energy was exploited to provide electric light and mobile phone charging for each household, and the impacts of these immediate tangible benefits upon acceptability of and adherence to the use of the intervention are being measured.

**Discussion:**

This study will be the first to evaluate whether the principle of solar-powered mass mosquito trapping could be an effective tool for elimination of malaria. If proven to be effective, this novel approach to malaria control would be a valuable addition to the existing strategies of long-lasting insecticide-treated nets and case management. Sociological studies provide a knowledge base for understanding the usage of this novel tool.

**Trial registration:**

Trialregister.nl: NTR3496 – SolarMal. Registered on 20 June 2012.

**Electronic supplementary material:**

The online version of this article (doi:10.1186/s13063-016-1469-z) contains supplementary material, which is available to authorized users.

## Background

Significant reductions in malaria infections and mortality since the year 2000 are associated with increased coverage of vector control interventions such as long-lasting insecticidal nets (LLINs) and indoor residual spraying (IRS), as well as improved availability and access to preventive therapies, diagnosis and treatment [1]. However, the development and spread of insecticide resistance and the occurrence of residual malaria transmission outdoors and in the early evening threaten the long-term sustainability of current tools for malaria vector control. This necessitates the development of new alternatives, particularly as many regions move towards malaria elimination [2, 3]. In 2013 an estimated 538,000 people lost their lives due to malaria with 90 % of those deaths occurring in the WHO African Region [4]; a region where millions of dollars of malaria-associated economic losses are suffered every year [5]. With the addition of new tools for malaria control that could reduce household spending on malaria-associated expenses, millions of people could potentially escape the cycle of poverty and disease. Estimates show that for each dollar spent to control malaria, up to 60 USD worth of benefits could be gained for the overall well-being of a society in the sub-Saharan Africa region [6].

Studies to characterise the components of human odour which are attractive to host-seeking *Anopheles gambiae s.s.* have led to the identification of a large number of compounds [7–9] which, at appropriate concentrations, can be combined to create synthetic mosquito lures that mimic a human host [8, 10]. These lures can remain attractive to mosquitoes even after a year of use [11]. Synthetic lures can be placed in counterflow trapping systems and used to lure and capture host-seeking mosquitoes both inside and outside houses [12–14]. By capturing mosquitoes outdoors, rates of mosquito house entry can be lowered by between 33–80 % under semi-field conditions [10, 13] and by 50 % in the field [15]. It is anticipated that above a certain threshold level of trap coverage, traps could be used to effectively reduce *Anopheles gambiae s.l.* and *Anopheles funestus* populations enough to lower the entomological inoculation rate (EIR) to a level at which malaria transmission cannot be sustained [16]. The principle of mass trapping for the control of tsetse flies has already been demonstrated in several African countries [17, 18], and we expect that this principle can also be applied to malaria vectors.

The Asembo Bay area of western Kenya was one of the first regions in sub-Saharan Africa to receive insecticide-treated bed nets (ITNs) as part of a trial in the mid-1990s [19–21], but despite increasing population coverage of ITNs since 2000, as well as provision of artemether-lumefantrine, intermittent IRS and presumptive treatment in pregnancy, malaria remains prevalent in western Kenya [22, 23]. The history of sustained vector control interventions as well as extensive prior understanding of malaria and malaria interventions in the Lake Victoria region of Kenya mean that this setting is ideal for a study investigating the efficacy of odour-baited traps combined with LLINs and case management for malaria control.

In this rural region of Kenya, few residential buildings are connected to the main electrical grid, and most households light their homes using kerosene tin lamps. The requirement of electrical energy to power the fan inside the odour-baited trap prompted the decision to integrate the mosquito trapping systems into a solar-home system, henceforth referred to as a solar-powered mosquito trapping system (SMoTS). The SMoTS includes two light emitting diode (LED) lights and a mobile phone charging port in addition to the odour-baited mosquito trap. These additional, immediate, private benefits of the system were expected to increase usability and improve adherence to the public health intervention, which requires the sustained participation of residents [24].

Here we describe the study design and methods used by the SolarMal project to test this intervention on Rusinga Island, western Kenya (the WHO Trial Registration Data Set is given in Additional file 1, and a protocol checklist is given in Additional file 2). The SolarMal project is the first trial to measure the efficacy of this novel approach to malaria vector control. A stepped wedge cluster-randomised approach was applied to the intervention rollout so that the intervention coverage gradually increased from no coverage to coverage of all eligible households over the course of 24 months. During the course of this study we aimed to determine whether the addition of daily removal trapping of malaria mosquitoes to the Kenyan national malaria control strategy (LLINs plus case management) will lead to elimination of malaria from Rusinga Island, western Kenya.

### Study objectives

#### Primary objective

The primary objective is to measure the effect of mass mosquito trapping on clinical malaria incidence, measured as fever plus positive rapid diagnostic test (RDT) result.

#### Secondary objectives

The medical objectives are as follows (all outcome measures include contemporaneous comparison of intervened with non-intervened areas, as well as before-and-after measures of intervened areas compared with baseline):

- To determine the impact of mass mosquito trapping on malaria prevalence measured by RDT, with subsequent high-resolution melting PCR used to estimate the prevalence of sub-patent malaria
- To calculate differences in both measured and reported all-cause fevers following the introduction of odour-baited traps

The entomological objectives are as follows (all outcome measures include contemporaneous comparison of intervened with non-intervened areas, as well as before-and-after measures of intervened areas compared with baseline):

- To assess whether the mass trapping of mosquitoes reduces the population density of malaria vectors on Rusinga Island
- To determine whether the mass distribution of odour-baited mosquito traps leads to changes in mosquito species composition
- To record changes in entomological inoculation rate associated with implementation of the intervention
- To compare mosquito densities and species composition indoors and outdoors

The sociological objectives are as follows:

- To determine the behavioural, socio-cultural and organisational factors that influence the effective and sustainable use of SMoTS
- To foster learning relevant to adapting the implementation and sustainability strategy as an integral component of the intervention
- To understand how the introduction and use of SMoTS affects and/or is affected by the use of other malaria control interventions

## Methods/design

### Study area and participant eligibility

The study took place on Rusinga Island, western Kenya, an island that is located approximately 75 km southwest of the city of Kisumu and has a surface area of around 44 km^2^. Research activities are conducted through the Thomas Odhiambo Campus of the International Centre of Insect Physiology and Ecology (*icipe*) in Mbita Point, located 2 km from Rusinga Island. In a population census conducted by the project in May 2012, the total population of the island was 23,337 people, living in 4062 households [25]. The majority of the population belongs to the Luo ethnic group, and Dholuo is the main language spoken by residents. A household (locally referred to as a homestead or *dala*) may comprise more than one house. The primary occupations of people are fishing in Lake Victoria and small-scale farming. The climate is tropical with a long rainy season typically occurring from February to May with a shorter rainy season from October to November. Malaria is typically endemic in this region, and transmission occurs throughout the year [22, 26].

**Fig. 1:**
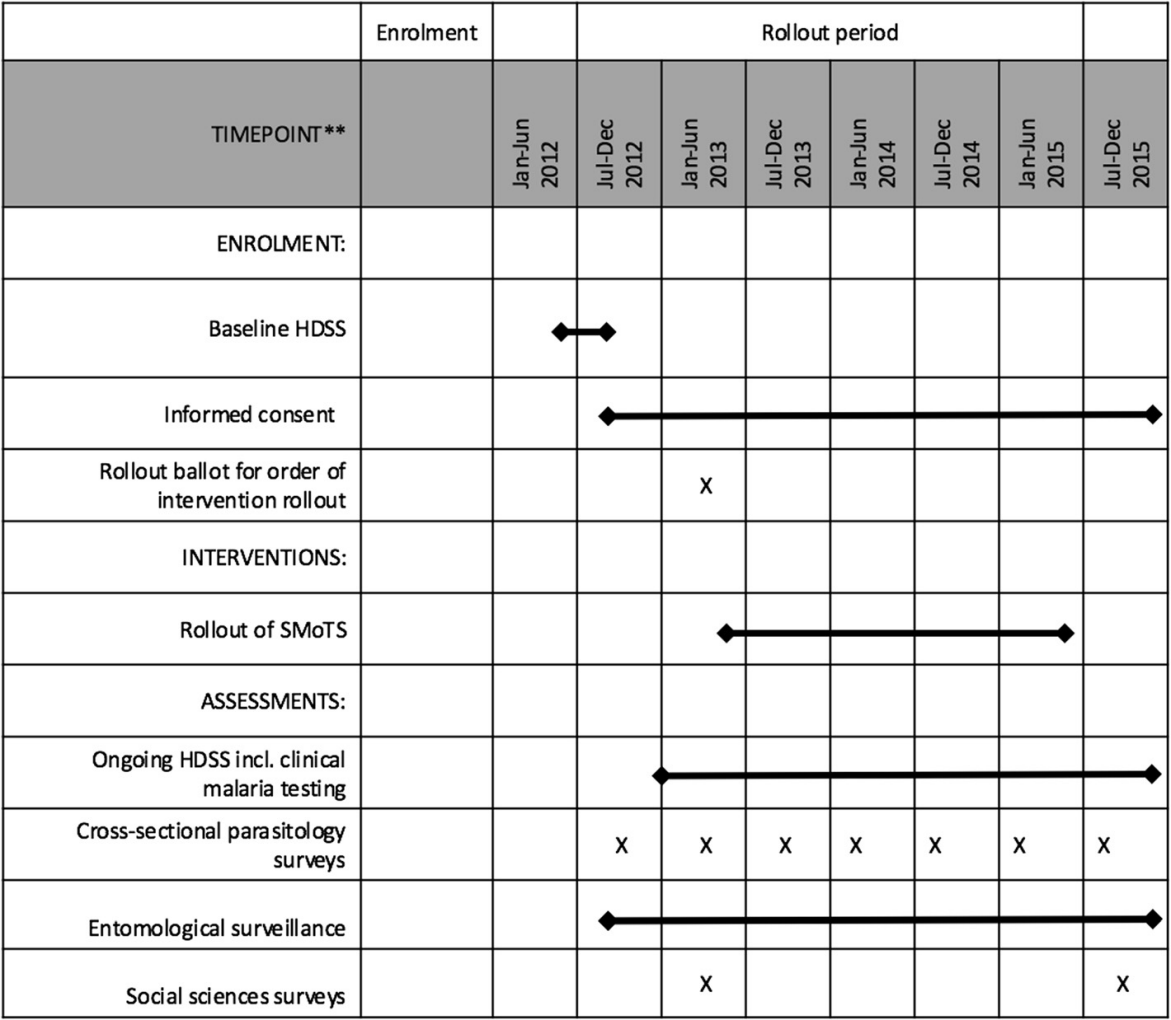
SPIRIT flowchart for the SolarMal project showing the timing of enrolment, interventions and assessments

**Fig. 2:**
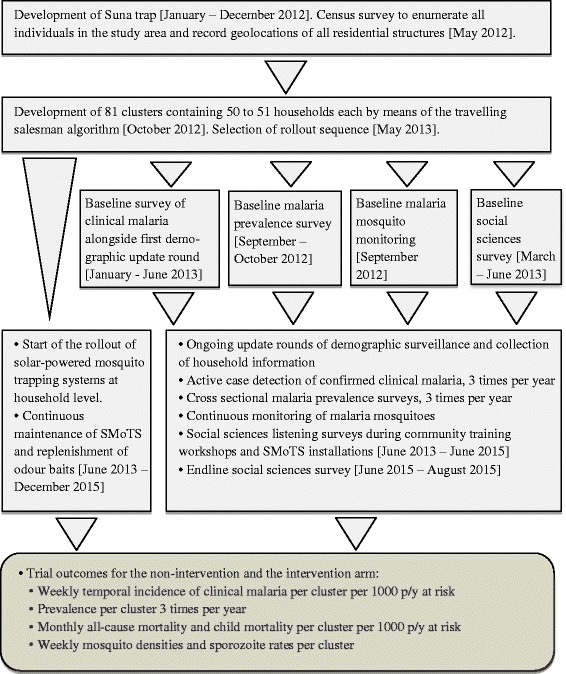
Diagram showing the workflow of the SolarMal project from planning stages to completion of intervention rollout

All households and residents of Rusinga Island were eligible for inclusion in the study with recruitment commencing in June 2012 and continuing until November 2015 (Figs. 1 and 2). The assignment of households to clusters and metaclusters (see the section on study design below) was completed in May 2013, and any household constructed before this point was eligible to receive an odour-baited trapping system. Households constructed after this time were eligible to participate in the health and demographic surveillance, parasitological, entomological and sociological studies, but were no longer eligible to receive a SMoTS. As the rollout of SMoTS took two years to complete and we did not return to previously installed project clusters to install SMoTS in new households, limiting SMoTS to those households already built at the start of the rollout meant that the intervention coverage levels were consistent across the island. Had SMoTS been installed in all households (including those built after the start of the rollout) there would have been a greater coverage of SMoTS in those clusters which received the intervention later in the two year rollout process.

In order for the results of the intervention to be generalisable across whole societies, all residents of the island were eligible for participation, regardless of age, gender, ethnicity, health status or whether they were natives of the island. For overall participation in the study and recruitment to health and demographic surveillance (HDSS) as well as malaria testing by RDT, individual written consent was provided by adults aged 18 years and older for themselves and for mature minors. For persons aged 13–17 years, individual assent was provided alongside written consent of an adult. For persons under 13 years of age, written parental consent was provided before recruitment to the study. All consent forms were in either English or Dholuo and were signed by the recruiter (a member of the project staff) and a witness. Informed verbal consent was provided by individuals or heads of household before participation in sociological and entomological studies, respectively. Participation in the study was voluntary, and all participants were free to withdraw at any time without giving a reason for their withdrawal. Unique identification numbers were assigned to preserve the anonymity of study participants.

Enumeration of the population and recruitment of participants were ongoing throughout the study period (i.e. from May 2012 until November 2015). Three rounds of HDSS took place during each year of the study, with recording of births and deaths as well as in- and out-migration across both arms of the study. A unique identification number was assigned to every individual, house and household recruited to the study during the HDSS.

The population of Rusinga was sensitised about project activities and findings in a number of ways throughout the course of the study. An initial community launch day was held on the island in August 2012 with the aim of informing community members about the project using song, dance, sketches and speeches. In order to ensure good communication between the project scientific staff and the study participants, a community advisory board (CAB) was established, including representatives of key groups of stakeholders. The CAB met formally four times each year to receive updates on project progress and plans from scientific staff and in turn to provide feedback from the community and to discuss project plans. Informal meetings between the CAB and project staff were also held whenever the need arose. In May 2013 a public balloting event was held where the sequence of the rollout was selected with the participation of community members [24]. Thereafter, weekly community training workshops were held to train each cluster of approximately 50 households in the maintenance of SMoTS.

### Intervention: solar-powered mosquito trapping systems (SMoTS)

The odour-baited traps (Suna traps) that were used during this intervention were developed in collaboration between Wageningen University and Research Centre (the Netherlands), the International Centre of Insect Physiology and Ecology (*icipe*, Kenya) and Biogents AG (Germany) [13]. The traps were baited with a blend of five organic attractants that mimic a human odour and lure mosquitoes towards the trap [10]. The blend of five chemicals was supplemented with a carbon dioxide mimic [27] in order to increase the attraction of malaria vectors to the trap. The odour baits were produced at the field site in Kenya by impregnating strips of nylon with each attractant at the appropriate concentration [28]. Baits were prepared in batches and stored at –20 °C to prevent the organic chemicals from volatising before use. Semi-field studies have shown that baits remain attractive to *An. gambiae* even after weekly use over 52 weeks [11]. During the course of the study odour baits were replaced in each intervened household by project field staff at 3-monthly intervals.

Previous studies have shown that a host-seeking mosquito can detect human or animal odours at distances of 50 m or more [29, 30], and we expect that the odour-baited Suna trap has a similar radius of attraction. Traps were suspended outside houses, beside the primary sleeping area with the fan section at 30 cm above the ground, a position that has previously been shown to result in the highest mosquito catch rates [13]. As described in the preceding background section, the requirement of electrical power for the trap meant that each SMoTS comprised an odour-baited mosquito trap, solar panel, battery, two LED lights, one mobile phone charging port and the associated electrical wiring.

During the course of the study each eligible household on Rusinga Island was offered one SMoTS. If a household comprised more than one residential structure (house), the project staff requested household members to reach a consensus agreement as to which house should have the SMoTS installed. If no consensus was reached, the SMoTS was not installed.

Two weeks prior to SMoTS installation in any given cluster, residents of the cluster were invited to attend a community training workshop held at a local community centre, such as a church or school building. During each training workshop study participants were reminded of the aims of the study and took part in question-and-answer sessions about malaria transmission and prevention. Demonstration SMoTS were used to show participants how the system operates and how to empty the trap of mosquitoes and clean it on a weekly basis [31]. Contact information for project-employed technicians was provided so that any technical faults in the systems could be reported and resolved promptly.

### Study design

The SolarMal trial used a stepped wedge cluster-randomised trial (SWCRT) design [32] where the intervention is allocated to geographically defined clusters in a randomised order until full coverage is achieved. This trial design is appropriate for a vector control intervention such as an odour-baited trap which is expected to have an impact that extends to an area beyond the house on which it is installed (spill-over effect). Replication of the intervention in multiple clusters while maintaining contemporaneous control areas can be achieved with a cluster-randomised trial (CRT) design, typically aiming to reduce infection at the individual level by targeting a whole community/area with the intervention. The stepped wedge design provides the opportunity of attaining area-wide coverage and group randomisation by the gradual crossover of all clusters to the intervention arm. In this way the effect of the intervention can be measured when used at a relatively small scale, up to mass coverage. Given the contemporaneous controls, the stepped wedge design also allows some ability to control for time.

### Randomising the intervention allocation

**Fig. 3:**
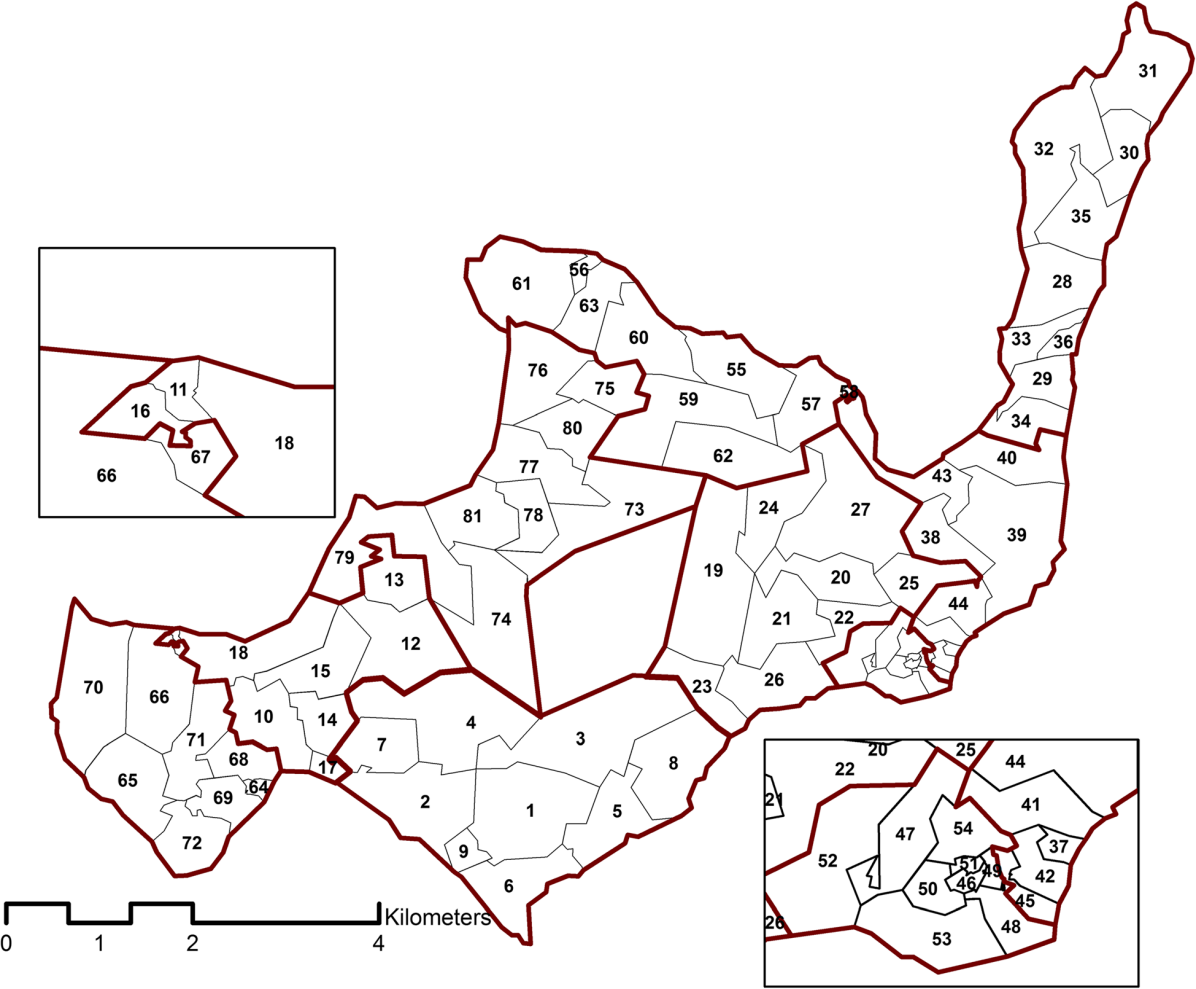
Rusinga Island with 81 project clusters, each containing 50–51 households, numbered consecutively in the order in which the SMoTS were installed. Metaclusters, each containing nine clusters, are outlined in *bold red lines*. Insets show close-up views of geographically smaller clusters in the northwest and southeast of the island. The blank space at the centre of the island is an uninhabited hill

Clusters of households were constructed by means of a travelling salesman algorithm whereby the shortest distance from one household to another was continually chosen, creating a cluster after every 50 or 51 households (see Fig. 3). The number of houses per cluster is expected to be large enough for measurement of the maximal intervention effect at the centre of the cluster, avoiding spill-over of mosquitoes from surrounding non-intervened areas resulting in sustained malaria transmission. The degree of protection among people living in households at the edges of clusters may be affected by mosquitoes from surrounding non-intervened areas; alternatively, intervened households located at cluster edges may exert an effect on mosquitoes in neighbouring areas which are yet to receive the intervention, as was observed during the early bed net studies [21]. Our clusters were expected to be large enough to include both substantial areas at the centre where the maximal intervention effect is obtained, and peripheral areas where spill-over of mosquitoes from surrounding non-intervened areas could be measured.

Computer simulations of possible rollout scenarios were made on the basis of a human susceptible-infected-susceptible transmission model [33]. A hierarchical design was selected and adopted as the rollout strategy for SolarMal. The design groups the 81 clusters of 50 or 51 households into nine larger areas, each referred to as a metacluster. Within every metacluster the intervention was subsequently introduced to each of nine clusters in a random order. Once the intervention had been applied to all clusters in one metacluster, the rollout moved randomly to the next metacluster; all clusters eventually received the intervention according to this SWCRT design. The nature of the intervention (visible trap outside the house and solar panel on the roof) meant that it was not possible to blind the intervention.

Prior to a community rollout ballot held in May 2013, a large number of possible rollout sequences were computer generated, each consisting of a distinct ordering of the 81 clusters [33]. Nine possible rollout sequences (each of which was compliant with statistical power calculations and requirements of the community) were presented by the researchers for blind selection by community members, with one sequence starting in each of the nine metaclusters and preserving independence among metaclusters. After placing a printed map of each sequence in a sealed, unmarked envelope and placing the nine envelopes into a box, one member of the community was chosen at random to draw an envelope and open it to reveal the order of the rollout which would be followed [24]. The selected SWCRT sequence is illustrated in Fig. 3.

### Demographic surveillance

During successive rounds of health and demographic surveillance (see Figs. 1 and 2 for schedules), records of the complete population of the island were maintained by door-to-door visits, collecting data using a tablet computer installed with data collection and management software (see the section on data entry and management below) [34]. Data were uploaded to the local server on a daily basis, creating a near real-time demographic database that subsequently served other parts of the project. A team of fieldworkers collected data simultaneously in all nine metaclusters on a daily basis. Over the course of 3 months all households and individuals were visited by fieldworkers to update demographic information. By conducting successive rounds of surveillance, data were available for each of the 81 clusters throughout the baseline and rollout period, thus providing information about both arms of the intervention for before-and-after and contemporaneous measures of intervention effect. The last survey before the start of the intervention rollout served as a baseline record of the population, and took place from January to June 2013. The location of all houses was recorded using a GPS built into the tablet computers.

### Measurement of malaria incidence and prevalence

Clinical malaria incidence was recorded during the routine HDSS surveillance of all individuals (three rounds of surveillance each year, see Figs. 1 and 2). During household visits residents were asked to report any fever in the previous 2 weeks, 2 days and at the time of the visit. If fever was reported to have occurred within 2 weeks of the visit, body temperature was measured using an in-ear thermometer (Braun™ IRT 3020). If the measured in-ear temperature was greater than 37.4 °C, the individual was tested for malaria using an RDT (*SD BIOLINE*™ Malaria Ag P.f/Pan *HRP*-*II*/pLDH). Any person with a positive RDT was provided with an appropriate dose of artemether-lumefantrine or referred to a local health clinic in the case of pregnancy, child under 6 months of age or severe symptoms. By collecting clinical malaria data continuously, information was available for baseline and throughout the course of the rollout for all 81 of the clusters.

In addition to the detection of malaria-associated fever within the HDSS, cross-sectional malaria prevalence surveys were carried out in a randomly selected 10 % of the study population three times per year (Figs. 1 and 2). All selected individuals were tested for malaria using an RDT, and a dry blood spot was also collected from each person as well as a measure of in-ear temperature. Validation of RDTs was performed using high-resolution melting PCR (HRM-PCR) [35] on a random sample of 200 dry blood spots from each round of surveillance. As for the HDSS and clinical malaria monitoring process, this data collection method allowed for measurement of malaria prevalence in all 81 clusters of the SWCRT at regular intervals throughout the course of the project.

### Entomological data collection and evaluation

Monitoring of mosquitoes began in September 2012 and continued until November 2015 (see Figs. 1 and 2). Sampling of mosquitoes at houses was performed using Mosquito Magnet-X® traps (American Biophysics Corporation, North Kingstown, RI, USA), baited with the same blend of five chemicals that are used for the intervention [10] and carbon dioxide produced by yeast and molasses fermentation [36]. For each round of sampling, 80 households were randomly selected with replacement from the active database maintained by the HDSS. In common with data collection in other arms of the project, random selection of households for entomological monitoring enabled measurement of entomological outcomes across the island throughout the duration of the SWCRT. Working four nights a week, ten houses were sampled every night with traps set at dusk (between 17:00 h and 18:00 h) and collected after dawn (between 07:00 h and 08:00 h). Each house was sampled once inside the house and once outside, with the inside/outside order randomised. The complete round of sampling took 4 weeks to complete, following which there was a 2-week period without sampling to make preparations for the next round. When a house had already been installed with a SMoTS, the Suna trap was disconnected during the two nights when the MM-X trap was used instead.

After collection of traps, mosquitoes were knocked down using a –20 °C freezer and identified to species group on the basis of morphology [37]. Specimens were separated and pooled by collection date, house of collection and inside/outside location, morphological species ID, sex and abdominal status. Pooled mosquitoes were stored in 80 % ethanol for subsequent molecular analyses: PCR for identification of *An. gambiae* s.l. complex and *An. funestus* s.l. complex [38, 39] and HRM-PCR for detection of *Plasmodium* DNA and blood meal analysis [35].

### Household and environmental data

Information was collected on variables that could have a direct or indirect effect on the association between the intervention and malaria infection or entomological outcomes. Every third health and demographic surveillance round incorporated a digital questionnaire for the collection of information about houses and households. Information about the construction materials used to build each house and the number of rooms was recorded, as well as the presence/absence of eaves and whether there were preventative measures taken against mosquitoes, such as LLINs and IRS and eave screening. Indicators of socio-economic status were also included in these update rounds, as was information on land and house ownership, house occupation and highest level of education of the head of household. Additionally, high-resolution satellite images were obtained to provide data on possible confounding environmental variables including the normalized difference vegetation index and a water accumulation index, also known as a topographic wetness index (TWI).

### Social sciences

A mixture of quantitative and qualitative approaches to social science data collection was used. Prior to the commencement of the intervention rollout, a structured questionnaire was completed with one adult male and one adult female in each of 204 randomly selected households (5 % of all households). The questionnaire was repeated with a new random selection of 5 % of households after completion of the rollout.

In addition to the structured questionnaires, listening surveys were conducted during each community training workshop to record trends in questions asked by community members over the course of the rollout. Listening surveys were performed during the installation of SMoTS in order to gauge initial reactions to the intervention. Data were also collected informally (i.e. observations, listening surveys, field notes) during other project community events such as an event held to launch the project on the island and the rollout ballot, among others. Throughout the course of the study, focus group discussions with specific stakeholders not only provided a useful tool for gathering information on community knowledge, attitudes and perceptions, but also helped the project to build links with the community.

Throughout the duration of the study, community members were able to contact a project community liaison officer and the solar technicians by phone in order to report technical faults in the SMoTS. A detailed record of phone calls was maintained by an on-site project manager, and these records were used to schedule maintenance activities as well as to understand how well the systems were performing over time. Intermittent spot checks were carried out one evening per week in 20 randomly selected households from a single metacluster to allow the field staff to monitor the performance of systems during the hours of darkness.

During the final phase of the project (December 2014–December 2015), interviews with key stakeholders and focus group discussions were used to develop and finalise a sustainability plan for the maintenance of SMoTS beyond 2015.

### Data entry and management

The collection and management of data was fully digitised, with all data entered by means of a tablet computer. The data collection was managed by the researchers, who operated independently from the project sponsor. Open Data Kit (ODK) [40] was used to build and conduct questionnaires, and data were uploaded daily to a secure local server. Demographic data were stored and then transferred to OpenHDS, a data management platform [34, 41]. New information was automatically incorporated into the demographic core database. This data management platform allowed for data cleaning immediately after upload to the server. To prevent duplication of ID codes in the system, the OpenHDS software generated a new unique ID for each individual, house and household as required. There were several built-in methods to prevent errors in data entry. Mostly, answers needed to be logical and were listed as multiple choice in the electronic questionnaires. For instance, a male cannot be recorded as having a pregnancy, and the age of a newborn cannot be more than one year.

A progress and quality monitoring plug-in for OpenHDS, SU2, used to ensure quality post hoc, was deployed in 2013 [42]. The programme, which automatically ran every night, provided the data manager with a report on operational statistics and inconsistencies in data collected the previous day. The SU2 software tracks which individuals and houses are visited on a daily basis and produces an up-to-date geodatabase of locations to visit for uploading to the tablet computer. Maps based on this information guided fieldworkers in navigating through their assigned area and recognising which houses and individuals still needed to be visited during a round of surveillance. The final trial database is stored at Wageningen University and Research Centre and is available to the entire research team.

### Power and sample size rationale

There is some controversy about power calculations for SWCRTs [43], and the power of our design depended on the correlation between observations on the same individuals at sequential HDSS visits. We could not determine the level of correlation from the single baseline enumeration visit. A lower bound for the minimum detectable effect size is therefore that of a single visit per person, occurring halfway through the rollout. Using previously published formulae, this approach was anticipated to have had 80 % power to detect approximately 52 % reduction in clinical incidence with an effective sample size of 7914 persons [44]. Analogous calculations for prevalence [44], using a baseline malaria prevalence of 23.7 % (RDT prevalence rate during the baseline survey for this project) and minimum sample size of 907 persons, suggested that a single prevalence survey should have had 80 % power to detect a 27 % reduction in prevalence. Six repeated surveys might have the power to detect effects as small as an 11 % reduction in prevalence, assuming that correlations between repeated observations were small.

### Analytical plan

The datasets included for the analysis comprise results of the HDSS, clinical malaria surveys, cross-sectional malaria prevalence surveys and monitoring of mosquito densities. Malaria fever incidence is the primary outcome of the trial. Data are included up to the end of the next month after full intervention coverage was attained. For analyses of parasitological as well as entomological outcomes, intervention status is classified week by week on the basis of whether traps had been installed in the cluster. The whole study cluster is classified as intervened or non-intervened based on whether installation was complete in that cluster by the end of the week. Clusters are excluded from analysis during weeks in which some, but not all, of the households were provided with the intervention (i.e. during the week in which installations took place in that cluster). For malaria prevalence and incidence, the numbers and proportions of positive RDT tests were summarised by week and arm of the trial. For mosquito densities, rates of anophelines collected per trapping night were presented by week and arm of the trial.

Following the SWCRT design described by Silkey et al. [33], an analytical plan was constructed. The primary analysis of the impact of the trial on clinical malaria incidence, prevalence and vector densities follows two measures of effect: a contemporaneous comparison comparing the outcomes in intervened clusters with the not yet intervened clusters and a comparison of the final results in intervened areas with the baseline status. Generalized linear mixed models (GLMMs) with a binomial distribution will be deployed to carry out significance testing against the null hypothesis of no effect using a likelihood ratio test. For mosquito densities a GLMM will be used with a Poisson distribution. For analysis of medical and entomological data, random effects will be used to allow for spatial effects (cluster) as well as effects of round of surveillance (time period within cluster). Final models will consider possible confounding effects on the relationship between the intervention effect and measured outcomes. The analytical plan did not include any interim analyses.

## Discussion

The long-term sustainability of malaria control achieved through the use of LLINs and case management with drugs is threatened by the development of insecticide and drug resistance. The SolarMal project has been designed to test for the first time whether mass trapping of mosquitoes can form a viable option for malaria control on Rusinga Island in Kenya, in addition to the already established LLIN plus curative strategy of the Kenyan National Malaria Control Programme (NMCP). The study took place in an area where LLIN coverage is high and drugs for case management are available and accessible.

The primary outcomes of the study will provide information about the efficacy of mass mosquito trapping on clinical malaria incidence, *Plasmodium* parasite prevalence, mosquito densities, EIR and sociological outcomes. A SWCRT design allows for before-and-after as well as contemporaneous measures of intervention effect, and clustering of the intervention permits measurement of a possible spill-over effect of traps into neighbouring non-intervened areas. Through gradual scale-up of intervention coverage over 2 years, with baseline measurements before the commencement of the rollout and at least 7 months of follow-up after completion of the rollout, an understanding of the time taken to achieve an impact through mass trapping will also be gained. By gathering data on multiple outcomes, i.e. human health and entomology, we anticipate that it will be possible to attribute an effect on malaria to the SMoTS intervention. Likewise, if the intervention is ineffective, it will be possible to offer explanations for this outcome. Understanding the mechanism behind a successful intervention will be vitally important in optimising the system for future scale-up and, in the instance of no observed effect, understanding this result will also allow improvements to the approach, which could lead to success in the future.

In addition to the anticipated impact on malaria, members of the study population were expected to immediately benefit through the electrical lighting and mobile phone charging facilities provided with the SMoTS. Electrical lighting is expected to reduce a reliance on kerosene that is typically used to light houses in this region. As the fumes emitted by burning kerosene are known to negatively affect the respiratory system [45], replacement of kerosene lamps by electric lights is likely to remove this health hazard. As well as removing health risks attributed to inhalation of kerosene fumes, the risk of fire and burns [46] is also reduced by providing electric indoor lighting. With a reduced daily expenditure on kerosene and mobile phone charging, the intervention should lead to financial savings and could lead to improved socio-economic status, which in turn may lead to other health improvements.

In order to ensure that risks to the population were minimised, the continued use of LLINs by all age groups was recommended at all community meetings and training sessions. Participation in the intervention did not affect the use of existing health facilities. The creation of a CAB facilitated regular exchanges of information between scientists, project field staff and the Rusinga Island community, and it is expected that some members of this board will remain actively involved in the maintenance of the SMoTS beyond the follow-up period of the study. By the completion of the rollout in mid-2015, the community were beginning to form groups to save money for the purpose of maintaining SMoTS beyond the research period. The provision of electrical lighting and mobile phone charging provides an incentive for users to keep the systems running, and links with Kenyan solar-home system providers are being made to ensure continuous provision of replacement components at prices which are affordable for low-income households. By working closely with the Kenyan Ministries of Health and Energy, the SolarMal project has formed a strong basis for continuing and expanding the use of SMoTS on Rusinga Island and elsewhere in the region. If the intervention is proven to be an effective tool for malaria control, researchers will work together with industry and policy makers to develop cost-effective, long-lasting and readily available malaria mosquito trapping systems for use in at-risk areas.

During the course of this study there was not a specific protocol for monitoring mosquito behavioural adaptations. Unlike well-established tools for mosquito control such as insecticide-treated bed nets and indoor residual spraying, there are no protocols for measuring behavioural adaptations to odour-baited traps. Future studies could measure the responses of field-caught mosquitoes from Rusinga Island to the odour baits and compare response rates with lab-reared mosquitoes, or with responses of mosquitoes from areas where odour-baited traps have never been used.

Additional studies and modelling of the impact of this trial will be required to extrapolate the findings of this study to other areas, including regions with different dominant vector species and routine control practices. It is anticipated that a scale-up of systems would follow a public-private model with investment from governments and non-governmental organizations (NGOs) as well as financial contributions by end-users. Scale-up would initially be focused in the East African region with exploratory studies in the Americas and Southeast Asia.

### Trial status

The rollout of SMoTS was complete at the time that the protocol manuscript was submitted for review by *Trials*. Recruitment of participants for continued HDSS and entomological monitoring of intervention efficacy was completed in November 2015 with a view to assessment of the long-term effects of SMoTS on malaria.

## Abbreviations

CAB, community advisory board; EIR, entomological inoculation rate; HDSS, health and demographic surveillance system; ITN, insecticide-treated bed net; KEMRI, Kenya Medical Research Institute; LLIN, long-lasting insecticidal net; NMCP, National Malaria Control Programme; ODK, open data kit; RDT, rapid diagnostic test kit; SMoTS, solar-powered mosquito trapping system(s); SWCRT, stepped wedge cluster-randomised trial; TWI, topographic wetness index; WHO, World Health Organization

## Additional files

Additional file 1: World Health Organization Trial Registration Data Set for the SolarMal Project. (DOCX 117 kb) Additional file 2: SPIRIT checklist for the SolarMal project, indicating which manuscript page contains each element of the study protocol. (DOC 122 kb)
