# Supplementary material for: Mass mosquito trapping for malaria control in western Kenya: study protocol for a stepped wedge cluster-randomised trial
Source: Trials. 2016 Jul 26;17:356. doi: 10.1186/s13063-016-1469-z (PMC4962350; doi:10.1186/s13063-016-1469-z)
Supplement: Additional file 1: — World Health Organization Trial Registration Data Set for the SolarMal Project. (DOCX 117 kb) [file 13063_2016_1469_MOESM1_ESM.docx]

Additional file 1: World Health Organization Trial Registration Data Set for the SolarMal Project.

| **Data Category** | **Information** |
| --- | --- |
| Primary registry and trial identifying number | The Dutch Trials Register/Nederlands Trial Registrer  www.trialregister.nl: NTR3496 – SolarMal |
| Date of registration in primary registry | 20 June, 2012 |
| Secondary identifying numbers | N/A |
| Source(s) of monetary or financial support | The COmON Foundation, the Netherlands via University Fund Wageningen |
| Primary sponsor | The COmON Foundation, the Netherlands via University Fund Wageningen |
| Secondary sponsor(s) | None |
| Contact for public queries | \| Erik Toussaint, [erik.toussaint@wur.nl](mailto:erik.toussaint@wur.nl), +31 6 51565949 \|  \|  \| \| --- \| --- \| --- \| |
| Contact for scientific queries | Willem Takken, willem.takken@wur.nl, +31 317 484652 |
| Public title | Solarmal: Solar energy for malaria elimination |
| Scientific title | Solar-powered odour-baited mass trapping for malaria elimination |
| Countries of recruitment | Kenya |
| Health condition(s) or problem(s) studied | Malaria |
| Intervention(s) | Solar-powered mosquito trapping systems |
| Key inclusion and exclusion criteria | All households and residents of Rusinga Island, western Kenya, are eligible for inclusion. |
| Study type | Stepped wedge cluster-randomised trial |
| Date of first enrolment | June 2012 |
| Target sample size | Mass coverage of all households on Rusinga Island (4,062 households, 23,337 individuals at the time of the initial enumeration). |
| Recruitment status | Recruitment complete in November 2015 |
| Primary outcome(s) | Clinical malaria incidence measured during an ongoing health and demographic surveillance system (fever + positive RDT) |
| Key secondary outcome(s) | Malaria prevalence during cross sectional surveys of 10% random selections of the population (defined as RDT positive irrespective of body temperature). Anopheline mosquito densities. |
